# Assessment on the adverse effects of Aminoglycosides and Flouroquinolone on sperm parameters and male reproductive tissue: A systematic review

**Published:** 2015-03

**Authors:** Arash Khaki

**Affiliations:** *Department of Pathology, Tabriz Branch, Islamic Azad University, Tabriz, Iran.*

**Keywords:** *Aminoglycosides*, *Fluoroquinolones*, *Sperm*, *Male reproductive tract*

## Abstract

**Background:**

Antibiotic therapies used in treatment of many diseases have adverse effects on fertility. This review analyzes previous comparative studies that surveyed the effects of two common groups of antibiotics on male fertility.

**Objective:**

To evaluate histo-pathological effects of fluoroquinolones and aminoglycosides on sperm parameters and male reproductive tissue.

**Materials and Methods:**

Articles about the effects of aminoglycosides and fluoroquinolones on male infertility, sperm parameters, male reproductive tissue, and spermatogenesis in English and Persian languages published on Google Scholar and PubMed databases from January 2000 to December 2013 were assessed. Randomized controlled trials (RCTs) assessing the effects of aminoglycosides or fluoroquinolones on sperm parameters, artificial insemination, and male reproductive tract or RCTs comparing aminoglycosides vs. fluoroquinolones were eligible for inclusion. For ascertaining the reliability of study, data were extracted independently and in duplicate by two investigators.

**Results:**

Sperm viability was decreased significantly with streptomycin, gentamicin, and neomycin (p<0.001). Sperm motility was decreased significantly with gentamicin and neomycin (p<0.05). Total sperm count was significantly decreased with ofloxacin, gentamicin, streptomycin, and neomycin (p<0.022). There was significant decrease in post-thawing motility with low dose and high dose of ciprofloxacin. Testis weight was decreased with gentamicin and ofloxacin significantly (p<0.011). There was significant decrease in seminal vesicle weight with gentamicin, neomycin, and ofloxacin (p<0.022). Furthermore, changes in epididymis weight, percentage of total apoptotic cells, and diameter of seminiferous tubule were significant with all drugs including streptomycin, gentamicin, neomycin, and ofloxacin (p<0.05).

**Conclusion:**

Streptomycin has less negative effects on cell’s apoptosis and sperm parameters as compared to other drugs. Gentamicin has more detrimental effects so lesser dosage and duration is recommended. Fluoroquinolones showed negative effects on testis tissue and sperm parameters. Ciprofloxacin has less adverse effects than gentamicin in artificial insemination.

## Introduction

Infertility is an inability to become pregnant after one year of unprotected intercourse. 15% of couples experience infertility. In general, some male infertility factors are anatomical factors (e.g. varicoceles, ductal obstructions or ejaculatory disorders) (1, 2). Furthermore, male infertility can be due to sperm parameters abnormalities; it is estimated that 40-90% of male infertility is due to defect in sperm production. A major proportion of male infertility is due to lack of sperm (azoospermia) or too little sperm (oligozoospermia). Other infertility factors related to sperm parameters include abnormal sperm morphology (tetratozoospermia) and insufficient sperm motility (athenozoospermia) (2, 3). 

According to statistical information of male infertility, sperm count has been declining over the last 50 years. Other factors leading to male infertility are cigarette smoking, infection, radiation, nutrition, reactive oxygen species, estrogens, heavy metals, scrotal temperature, sperm antibodies, anti-bacterial drugs, and some therapeutic drugs. Antibiotic therapy affects spermatogenesis and seminal parameters in human and animals. Some antibiotics have detrimental effects on spermatogenesis, sperm parameters and fertility; these effects have been reported with antibiotics, including nitrofurantoin, sulfasalazine, tetracycline derivatives, penicillin group, ampicillin, aminoglycosides (Gentamycin and neomycin) (1). 

Artificial insemination (AI) has been developing over the last 50 years worldwide used for almost all animal species (4). Addition of the animal origin component (yolk or egg) for semen freezing has a potential contamination of bacteria or mycoplasma. These micro-organisms especially bacteria in ejaculates can impair spermatozoa leading to defect in fertility (5, 6). Furthermore, the toxins released by these micro-organisms impair fertilization. It has been reported that semen and embryos generated by assisted reproductive techniques (ARTs) may be contaminated by several micro-organisms from local or systemic male reproductive tract infections of donor or during ARTs procedure. Hence, different procedures are used in order to remove contamination, including washing procedures, antibiotics, and enzymatic treatment, treatment by antibodies or ozone, photo inactivation, acidification and using of novel antivirals. 

Since ARTs is being used worldwide and antibiotics especially aminoglycosides (e.g. gentamicin and neomicin) and fluoroquinolones (e.g. ciprofloxacin, ofloxacin) have a developed usage in ARTs and many bacterial diseases, we regulated a systematic review in order to evaluate and then compare the effects of aminoglycosides and fluoroquinolones on fertility rate (7).

## Materials and methods

Systematic literature searches were assessed from English and Persian articles published on Google Scholar and PubMed databases from January 2000 to December 2013. The following keywords were used in order to search: “aminoglycosides”, “fluoroquinolones”, “male infertility”, “sperm parameters”, “male reproductive tract”, “spermatogenesis”, and “artificial insemination”. The articles were screened by reading the ‘title’ and thereafter the ‘abstract’, and ‘full-text’. Finally, 32 articles were included in this study, 7 articles that hadnt RCT were excluded of this research and dates of 25 articles used as references ranged from 1996-2013. 

Twenty five articles that contain RCT were eligible for this study from January 2000 to December 2013. To obtain additional data, a manual search was performed using the reference lists of included articles. Thereafter, effects of fluoroquinolones, aminoglycosides, and comparative study on both drug families were demonstrated in tables. In order to ascertain the validity of eligible randomized trials, two reviewers with Ph.D degree in reproduction medicine in Tabriz University of medical sciences and Islamic Azad University, Tabriz Branch, Iran were working separately and determined the year of publication, each of quantities mentioned in the article and comparative results.

## Results

**Study selection**

**Figure 1:**
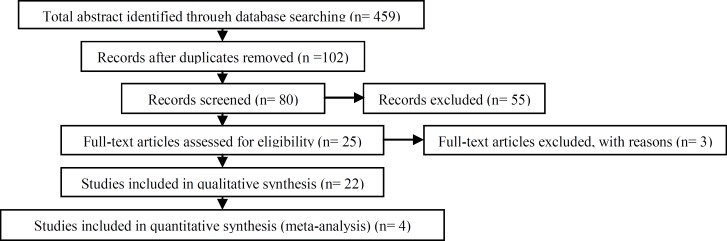
Flow diagram of study selection

Randomized controlled trials (RCTs) assessing the effects of aminoglycosides or fluoroquinolones on sperm parameters, AI, and male reproductive tract or RCTs comparing aminoglycosides versus fluoroquinolones using were eligible for inclusion (25 Articles), (Figure 1).

**Fluoroquinolones effects on male reproductive tract and sperm parameters**

One study showed apoptotic effect of ciprofloxacin as a quinolone is related to mitochondria pathways. Sperm cell toxicity is induced by mitochondrial pathway. Ciprofloxacin inhibited cell growth and induced apoptosis in certain eukaryotic cells (8). In a study, the effects of some quinolones were assessed on male infertility. After using ofloxacin, ciprofloxacin and perfloxacin, results demonstrated sperm count, motility and production as well as testicular lactate dehydrogenase (LDH-X) activity were decreased significantly. Ofloxacin effects were in a dose-dependent manner. Moreover, ofloxacin caused signiﬁcant increase in total serum acid phosphatase activity. Therefore, these results and histo-pathological changes demonstrated these drugs can cause testis dysfunction (9).

In a study, it was reported that there is a correlation between dose and treatment duration of ofloxacin and testicular toxicity in rat. Ofloxacin effect in high dose compared with low dose and in long-term was compared with short-term. Oﬂoxacin intake for 14 days (short time) at low or high dose showed non-signiﬁcant differences as compared with the control. However, oﬂoxacin intake for 28 days (long time) demonstrated changes in the estimated parameters mainly with high dose. Histo-pathological changes were focalin distribution, including sloughing, atrophy, degeneration, hypospermatogenesis and Leydig cell hyperplasia. Moreover, there was signiﬁcant negative correlation between body weights and epididymal sperm parameters with the dose and/or duration of the treatment (10).

Khaki *et al *assessed ciprofloxacin effect on testis apoptosis and sperm parameters in the rats; ciprofloxacin had toxicological effects on reproductive tract, such as decrease in the sperm concentration, motility and viability. Ciprofloxacin caused a significant decrease in the number of spermatogenic cells (spermatogonia, spermatocyte, spermatid and sperm) in the seminiferous tubules and with increase in intertubular spaces and veins congestion when compared with the control group. The number of TUNEL positive germ cells per tubule was increased; spermatogonia and spermatocytes were the main germ cells with TUNEL positive apoptosis (11). 

Using enrofloxacin causes adverse effects on reproductive system in male mice. With this drug, there is decrease in epididymal sperm count and motility. Furthermore, the number of abnormal spermatozoa was increased in the group receiving enrofloxacin. This drug was the reason of spermatogenesis disruption causing decreased sperm motility and morphological abnormalities (12). Electron microscopic study of testis tissue in mice receiving ciprofloxacin showed hyperchromatin nuclei of spermatocyt I, Sertoli cells and myoid, mitochondria vacuolation of spermatogonia and spermatocyte cells, increased the thickness of spermatid tail. Therefore, ciprofloxacin leads to marked decrease in fertility index and testicular weight in experimental group (13). Ciprofloxacin had cytotoxic effects on spermatocyt I cells, and cell death and consequently infertility (14). 

In a study histological and biochemical changes of low dose (LD) of ciprofloxacin (CPFX) were compared with these changes of high dose (HD) of CPFX. Higher numbers of Sertoli cells/ seminiferous tubule (ST) showed lipid-positive reactions in both low and high dose CPFX-treated animals. Results showed ciprofloxacin decreases carbohydrate ratio in spermatogonia and spermatocyte cells with both low and high dose of ciprofloxacin. In mice receiving ciprofloxacin, numbers of Leydig cells were decreased. The majority of Leydig cells had a dense periodic-acid-Schiff (PAS) reaction for both LD and HD of ciprofloxacin. 

Cytoplasmic lipid accumulation was also changed after ciprofloxacin administration. In CPFX groups, there was significant higher numbers of lipid-positive spermatogonia and spermatocyte cells. In ciprofloxacin groups, the spermatogenesis cell lineage exhibited high numbers of cells with SB-positive cytoplasm. In ciprofloxacin groups, high lipase-stained sites in the cytoplasm of the spermatogenesis cells were observed. Lipase enzyme increased in the cytoplasm of these cells. Elevated testicular alkaline phosphatase was the other finding in the test groups. Significantly increased ALP-positive cells (spermatogonia and spermatocyte) /ST were observed in CPFX-tested groups. Besides, the effects of ciprofloxacin were also assessed on hormonal levels; testosterone level was decreased significantly; the serum levels of LH and FSH in high-dose treated-animals were decreased significantly (15).

Adikwu Elias *et al *in a study have assessed effects of ciprofloxacin and perfloxacin on sperm parameters of male Guinea pigs. According to the results, ciprofloxacin made a non-significant change in the weight of the animals while perfloxacin significantly decreased in the weight of the animals. Ciprofloxacin and perfloxacin decrease testicular weight significantly dependent upon the duration of drug exposure. Significant decrease in sperm count after treatment with perfloxacin or ciprofloxacin was also observed (p<0.05). Decreased sperm motility was observed with ciprofloxacin and perfloxacin dependent on duration of treatment (p<0.05). Ciprofloxacin and perfloxacin caused significant decrease in serum testosterone level compared with the control group dependent upon treatment duration. 

These drugs increased significantly sperm primordial cell in comparison with control group time dependently (16). In a study, ciprofloxacin effect was assessed on testicular tissue of male guinea pig. Ciprofloxacin caused decrease in testicular weight dependent upon time and dose of ciprofloxacin. Besides, ciprofloxacin decreased sperm count of male guinea pig dose and time dependently. Sperm morphology was decreased with this drug dependent on time and dose. There was a significant decrease in testosterone level with ciprofloxacin time and dose dependently (17). 

In a study by Osawe and Farombi, the modulation effect of *Moringa oleifera* leaves on induced oxidative stress of ciprofloxacin in testis and semen of rats was assessed. Biochemical parameters were evaluated in this study, such as malondialdehyde (MDA), hydrogen peroxide (H_2_O_2_), reduced glutathione (GSH) and activities of glutathione-S-transferase (GST), glutathione peroxidase (GPX), superoxide dismutase (SOD) and catalase (CAT); furthermore, testicular sperm number (TSN), daily sperm production (DSP) and sperm morphology were evaluated. Compromised tissue membrane integrity was estimated by lactate dehydrogenase (LDH) and gamma glutamyl transferase activities (GGT). Increased H_2_O_2_ and MDA levels and also decreased GSH, GST, GPX and SOD activities demonstrated induced oxidative stress by ciprofloxacin. Ciprofloxacin significantly caused elevation of GGT activities in both testis and semen and elevation of LDH in testis only. Ciprofloxacin decreased TSN and DSP. *Moringa oleifera* leaves modulated these changes due to their antioxidant properties (18). 

In a study, it has been shown marbofloxacin has a transient detrimental effect on sperm motility in goat buck. At the beginning of study, marbofloxacin caused significant decrease in sperm motility; however, at the end of study this effect was modulated (19). A survey was done in order to evaluate the ciprofloxacin effect on sperm DNA damage, fertility potential and early embryonic development in NMRI mice. This study showed in CPFX group, fertilization and two-cell embryo rates were significantly lower than of those in the control group (p<0.001). The rate of two-cell embryo with LD of CPFX was similar to control group. 

However, a significant increase in percentage of arrested embryos type I, II and III was observed in CPFX group as compared with control group. Arrested embryo type 1 was more frequent than the other arrested type in treated group. Furthermore, after culturing for 120 hr, there was higher percentage of blastocyst in control group in comparison with CPFX-treated group (p<0.001). CPFX caused DNA damage; significant higher level of abnormal single-stranded sperm DNA in CPFX-treated mice (p<0.05) for both LD and HD of CPFX. Significant higher percentage of sperms with protamine deficiency was observed in both LD and HD of CPFX (p<0.05); there were significant differences between HD and LD (20). All significant administration changes were summarized in table I.

**Results of comparative studies on the effects of aminoglycosides and fluoroquiolone on spermatogenesis and male reproductive tissue**

In a controlled randomized clinical trial, the effects of fluoroquinolones (ofloxacin) and aminoglycosides (gentamicin, neomycin, streptomycin) on testis apoptosis by TUNEL assay were assessed. The results showed that streptomycin induced less apoptotic germ cells compared to the other drugs, but it induced higher apoptotic cells compared to the control group. The highest apoptotic rate was related to ofloxacin (Table II) (21). 

In another study by Khaki *et al* the effects of gentamicin and ofloxacin on testis tissue were assessed under light and transmission electron microscope. In the gentamicin group, abnormal space in neighbor Sertoli cells, disappearing the cristae of mitochondria, presence of lysosomes in Sertoli cells, the heterochromatin nucleus of myoid cells were seen, but in the ofloxacin group vacuolation in mitochondria and increasing in the number of vacuoles in primary spermatocyte, fragmentation of nucleus in primary spermatocyte, increased germ cell degeneration; condensation of germ cell nuclei; heterochromatin nucleus of spermatogonia; dilation of endoplasmic reticulum system, and first stage of damage cell were observed. The testosterone level was compared between two interventional groups which is shown in table II (22). 

In one study, the effects of some antibiotics, including gentamicin, ofloxacin, neomycin and streptomycin were assessed on testis tissue and sperm parameters. Gentamicin and ofloxacin significantly decreased the weights of epididymis, testis and seminal vesicles. Therefore, it seems these antibiotics have toxic effects on male reproductive system. However, using streptomycin and neomycin did not decrease these weights. Sperm viability and count were decreased in all experimental groups; sperm motility was significantly decreased in the groups receiving gentamicin and neomycin. Using gentamicin, streptomycin, neomycin, and ofloxacin caused increased amount of apoptotic germ cells. According to the results of this experiment, aminoglycosides (gentamicin, neomycin, streptomycin) and flouroquinolones (ofloxacin) have adverse effects on sperm parameters and male reproductive system. However, these effects were fewer in the group receiving streptomycin in comparison to the other drugs (23). In a study that assessed the effectiveness of gentamicin and ciprofloxacin on camels’ semen extender, LD and HD of gentamicin and ciprofloxacin were evaluated and results were showed in table III.

Freezed semen doses without adding antibiotics have a broad spectrum of gram positive bacteria such as *Staphylococcus aureus, Staphylococcus epidermis* and *bacillus species *and gram negative species such as *Escherichia coli* and *proteus *species*. *Results showed addition of antibiotics does not have an immediate significant effect on sperm motility after addition of extender except for high dose of gentamicin (40 mg) that was a non-significant decrease (p>0.05).

Gentamicin decreased sperm motility and velocity, so it does not improve sperm motility in semen contaminated with bacteria. According to this study, the effects of low dose of gentamicin and ciprofloxacin were compared with high dose of them, there were no significant changes in sperm motility except for high dose of gentamicin that caused non-significant decreased in sperm motility (51±4.9%) as compared with sperm motility (59±4.3%) of low dose of gentamicin. 

Therefore, it is better to avoid using of high dose of gentamicin in intra uterine insemination (IUI). Gentamicin does not have any improving effect on sperm motility and velocity in bacterial-contaminated semen. Furthermore, ciprofloxacin didn’t have any significant effect on sperm motility and velocity. After equilibration period, gentamicin in high dose also decreased sperm motility and velocity when compared to the other groups. Post-thawing motility in high dose of ciprofloxacin group was higher than the other study groups; then higher sperm motility was for low dose of ciprofloxacin group. However, there was no significant change in post-thawing motility for both low and high doses of gentamicin. Acrosomal integrity was higher in the treated groups when compared to the control group. It seems high dose of ciprofloxacin is a good choice for contaminated semen due to having no negative effect on post-thawing motility and acrosomal integrity (Table III) (24). 

The effects of streptomycin and ofloxacin were also evaluated on apoptosis of rat’s Leydig cells. The results demonstrated the proportion of Leydig cells undergone apoptosis in streptomycin group was less than the other groups, so streptomycin is a better treatment with lower side effect (23). All comparative changes between fluoroquinolones and aminoglycosides are shown in tables II, III.

**Aminoglycoside effects on male reproductive tract and sperm parameters**

According to the researches on aminoglycosides, gentamicin induces oxidative stress in male reproductive tract and causes spermatogenesis damage. The effects of some doses of gentamicin 3 and 5 mg/kg in 1 and 35 days were compared. Findings demonstrated each dose of gentamicin decreased the seminal vesicle weight. The daily abnormal spermatid production was increased in a dependent-dose manner. The sperm count decreased at both doses. Furthermore, sperm motility decreased and sperm abnormality increased at high doses of gentamicin. Some structural changes were observed with high dose of gentamicin, including nuclear pyknosis, athrophic changes in a few tubules, sloughing of seminiferous epithelium, gaps in the epithelium, and tubular shrinkage. 

Besides, decreased activities of three enzymatic antioxidants, including SOD, catalase, GPx, and also ascorbic acid were observed in a dose-dependent manner. Increased thiobarbituric acid reactive demonstrates increased lipid peroxidation in the testis (25). In one study, it was observed that administration of 50 mg/kg/day gentamicin caused to increase the apoptotic cells percentage. In gentamicin group, the percentage of apoptotic cells was 22.11±1.11 while this amount was 6±2.11 in the control group (26).

In another study on gentamicin’s toxicity on rat sperm, cauda epididymal sperm reserves (CESR) was decreased after gentamicin administration significantly. Sperm motility was decreased following gentamicin administration (27). Nouri *et al* evaluated the protective effects of *Carrot Seed Extract* on spermatogenesis and cauda epididymal sperm reserves in gentamicin treated rats. Photomicrograph of testis showed, in group receiving gentamicin, seminiferous epithelial layers were decreased. Gentamicin caused a significant reduction in CESR; however, Carrot seed extract could elevate CESR (p<0.05). Although in hormonal levels, there were no significant differences for FSH level in all groups, but gentamicin reduced the LH level (28).

Akondi *et al *evaluated the effects of *Rutin* and *Naringin*on induced testicular oxidative stress by gentamicin and biochemical parametes in male wistar albino rats. Results demonstrated gentamicin produces increase in MDA levels (p<0.001) dose depenedently but decreases SOD and catalase levels. Sperm count, motility and viability also affected by gentamicin. There was decrease in the sperm count and reduction in percentages of progressively motile and viable spermatozoa. Normal testicular tissue was disturbed by gentamycin. Treated groups showed improved testicular tissue (29). Alp *et al* in a study assessed the effects of some antibiotics on testicular tissue and semen quality in rats; in this study the effect of streptomycin also was evaluated. Streptomycin had detrimental effects on the testicular biopsy score and spermatozoon head morphology, but had positive effects on the other spermatologic traits (30). 

Price *et al* assessed the effects of gentamicin on stallion semen. At lower temperature (5^o^C), stored semen did not need to add gentamicin while at higher temperature (15^o^C), it was necessary to add gentamicin in order to maintain sperm quality. However, addition of gentamicin just was necessary for maintaining more than two days (31). Aurich and Spergser assessed the effect of gentamicin on cooled-stored stallion spermatozoa contaminated with common bacteria; they showed gentamicin cannot modify detrimental effects of bacteria on sperm quality. Moreover, gentamicin induces functional defect of spermatozoa. Therefore, gentamicin was not a good choice in AI in this study (32). The results are shown in table IV.

**Table I:** Histo-pathological and sperm parameters changes reported with Fluoroquinolones

**Fluoroquinolones**	**Histopathological and biochemical effects**	**The effects on the sperm parameters and spermatognesis**	**Reference**
**Ciprofloxacin**			
	Sperm cell toxicity Inhibition cell growth	Reduction in sperm motility, production and count*	8
	Apoptosis in certain eukaryotic cells by mitochondrial pathway	Reduced sperm count and motility*	9
	Decrease in testicular LDH-X activity * Significant decrease in diameter of the seminiferous tubule * Significantly increased in vein diameter* Significant decrease in testis, epididymis and seminal vesicle weight*	Declined sperm viability**	11
	Hyperchromatin nuclei of spermatocyt I and sertoli cells and myoid Vacuolation of mitochondria of spermatogonia and spermatocyts cells increasing the thickness of spermatid tail	Decrease in the number of spermatogenic cells in seminiferous tubules*	14
	Marked decrease in fertility index and testicular weight, Dense PAS reaction in Leydig cell* Decreased numbers of Leydig cellsof connective tissue* Higher numbers of lipid-positive Leydig cells, spermatogonia andspermatocyte cells per ST* Significantly higher numbers of Leydig cells/mm2 with ALP-positive areas* Higher numbers of ALP-positive per streptomycin * Significantly decreased testosterone level* Significantly decreased serum levels of FSH, LH in high dose-treated animals*	Apoptosis in spermatogonia and spermatocytes by TUNEL method	15
	Significantly decreased testosterone and increased sperm primordial cells time-dependently* Decrease in testis weigh dependent on time in male guinea pig*	Decrease in the number of spermatogonia and spermatocyte cells (PAS reaction)*	16
	Decreased testicular weight dependent on both dose and time(HD)* Increased n sperm debris dependent on time and dose* Increased sperm morphology changes time-and dose-dependently*	Higher numbers of spermatogonia and spermatocyte cells per ST*	17
	Significant decrease in SOD (Unit/ mgprotein)* Significant decrease in GST (Unit/ gtissue), GPX (Unit/ gtissue) and SOD (Unit/ gtissue)*	Decreased sperm motility time-dependently*	18
	Significant decrease in the number and percentage of oocytes, fertilized oocytes, embryos (blastocysts) and arrest type I, Arrest type II, and Arrest type III with HD and LD dose of CPFX** Significant decrease in embryo two cell with HD** Significant increase in Groups Positive Acridine Orange staining (%) and Positive Aniline Blue staining (%) (DNA integrity and chromatin quality) in HD and LD*; with significant decrease between HD and LD in Positive Aniline Blue staining*	Decreased sperm count time-and dose- dependently*	20
**Perfloxacin**			
	decrease in testicular LDH-X activity * increased sperm primordial cells time-dependently*	Reduction in sperm motility, count and production*	9
	decrease in testis weigh dependent on time in male guinea pig* decrease in body weight in long-time treatment*	Reduction in sperm motility, count and production*	16
**Ofloxacin**			
	signiﬁcant increase in total serum acid phosphatase activity* decrease in testicular LDH-X activity *	Reduction in sperm motility, count and production *	9
	decrease in body weight in long time treatment with both high and low doses* decrease in absolute testis weight (g) in long time treatment with both low and low doses* significant decrease in testosterone level, Curve linear velocity, Linear velocity, Linearity index and Sperm normal forms with high dose in long time*	Decreased sperm count and motility in long time for both high and low doses*	10
**Enrofloxacin**			
	Cytoplasmic vacuolation of Sertoli cells impaired spermatogenesis Nearly complete spermatogenic arrest disorganization and sloughing of germ cells and morphological abnormalities	Decreased sperm motility	12

* = p<0.05

**Table II:** Results of comparative study on fluoroquinolones and aminoglycosides on male reproductive tissue and biochemical parameters

**Histopathological effect**		**testis apoptosis by TUNEL assay**	**Testis weight (gr)**	**seminal vesicle weight (gr)**		**Epididymis weight (gr)**	**Percentage of total apoptotic cells (spermatogonia and spermatocytes)**	**Apoptosis of leydig cells**	**Diameter of seminiferous tubule (µm)**	**Serum testosterone level (ng/ml)**
Control groups		7.3 ±2.41	1.53±0.03	0.55±0.016		0.30±0.025	7.3±0.762	1.01±0.41	385.3±0.1	3.6 ±0.13
Aminoglycosides										
	Gentamicin	24.15±10.17	1.24±0.03*	0.19±0.009*	0.20±0.038*		24.15±3.216*	---------	282.3±0.1*	1.4±0.06*
	Streptomycin	15.15±11.14	1.48±0.03	0.52±0.009	0.28±0.057*		15.15±3.523*	2.15±11.14	292±0.8*	---------
	Neomycin	25.15±9.11	1.44±0.03	0.21±0.009*	0.22±0.057*		25.15±2.881*	---------	279.3±0.05*	---------
Comparative results between aminoglycosides		Streptomycin has the least apoptotic cells	Significant decrease with Gentamicin *	Streptomycin is with less changes	the effect of streptomycin is less than the other drugs		The least differences were seen with streptomycin as compared with the control group	---------	Streptomycin effect is lower than other drugs	---------
Fluoroquinolones Ofloxacin		Apoptotic rate of 34.15±8.17	decreased testis weight significantly 1.35±0.03*	0.20±0.006*	0.20±0.038*		34.15±2.584*	6.15±8.17	272+0.9*	1.1±0.04*
Comparative results		Less apoptotic rate with streptomycin but Higher apoptotic rate with ofloxacin	All drugs decrease testis weight. Neomycin and Streptomycin are with less changes, so these drugs are with less detrimental effect on testis weight	Streptomycin is with less change than other drugs	Streptomycin effect on epididymis weight is less than other drugs while Gentamicin and ofloxacin have more adverse effect		Ofloxacinhas most percentage of total apoptotic cells while streptomycin has least apoptotic cells	Streptomycin has lower apoptotic leydig cell	Significant decrease with Ofloxacin, less decrease with streptomycin	Significant decrease with Ofloxacin
References		21	23	23	23		23	23	23	22

* Signiﬁcant difference compared with controls (p<0.05)

** Signiﬁcant difference compared with controls (p<0.001)

*** Signiﬁcant difference compared with controls (p<0.01)

**Table III:** Results of comparative study on fluoroquinolones and aminoglycosides on the sperm parameters and AI

**Sperm parameters ** **and AI results**			**Total sperm count** **No. of sperm/ rat** * **06**	**Sperm motility** **(%)**	**Sperm viability** **(%)**	**Motility % after dilution (0 min)**	**Motility** **% after equilibration (240 min)**		**Post-thawing** **% motility**	**Post-thawing detached acrosome %**
Control groups			57±0.20	48.4±2.03	79.2±3.40	59±3.2	57±3.3		23.3±1.1	7.3±0.7
Aminoglycosides										
	Gentamicin		30±0.260*	18.8±0.85*	40.9±1.08**	Gentamicin(40 µg/ml) 51±4.9 Gentamicin (20 µg/ml) 59±4.3	Gentamicin(40 µg/ml) 42±6.7 Gentamicin (20 µg/ml) 52±6.8		Gentamicin(40 µg/ml) 20±1.3 Gentamicin (20 µg/ml) 21.7±1.1	Gentamicin (40 µg/ml) 8.5±1.1 Gentamicin (20 µg/ml) 11±2.3
	Streptomycin		34±0.28*	50.4±1.60	45.6±1.75**	---------	---------		---------	---------
	Neomycin		21±0.19*	34.2±0.92*	28.6±1.06**	---------	---------		---------	---------
Comparative results between aminoglycosides			(Streptomycin is with least effect, but Neomycin is with most adverse effect).	(Neomycin has the most adverse effect while Streptomycin has a non-significant adverse effect).	(Neomycin has more adverse effect, but streptomycin has 1less adverse effect)	(High dose of Gentamicin decreased sperm motility)	(Decrease in sperm motility with HD of Gentamicin)		There is no-significant decrease in motility, (No significant differences between HD and LD of Gentamicin and control group)	Acrosomal integrity higher than control group
Fluoroquinolones										
		Ofloxacin	12±0.27*	48.6±1.80	23.3±1.27**	---------	---------	---------		---------
		Ciprofloxacin				(400 µg/ml) 59±4.5 (200 µg/ml) 60±4.6 No marked changes were seen	(400 µg/ml) 57±5.3 (200 µg/ml) 56±6.5 No significant changes with HD and LD	(400 µg/ml) 38.3±5.9*** (200 µg/ml) 28.3±1.1*** Significant increase in motility with HD and LD		(400 µg/ml) 9.6±1.7 (200 µg/ml) 9±0.8 Acrosomal integrity higher than control group
Comparative results between aminoglycosides and fluoroquinolones			Ofloxacin has more detrimental effect, but streptomycin has less adverse effect	Gentamicin has more adverse effect; No significant changes were seenwith ofloxacinand gentamycin	Ofloxacin has more adverse effect, but streptomycin has less adverse effect	High dose of Gentamicin should be avoided	High dose of Gentamicin should be avoided	The highest sperm motility for HD of Ciprofloxacin and then LD of Ciprofloxacin rather than control group***		Nearly similar effect on acrosomal integrity
References			23	23	23	24	24	24		24

* Signiﬁcant difference compared with controls (p<0.05)

** Signiﬁcant difference compared with controls (p<0.001)

*** Signiﬁcant difference compared with controls (p<0.01)

**Table IV:** The effects of aminoglycosides (gentamicin) on male reproductive tissue and sperm parameters

**Histopathological effect**	**Aminoglycosides (gentamicin)**	**Reference**
Testis weight	High dose of GS decrease testis weight significantly*	25
Seminal vesicle weight	Significant decrease in long time treatment for any dosage of Gentamicin *	25
DSP (Daily sperm production)*10^6^	Significant decrease in high dose of Gentamicin *	25
Daily abnormal spermatid production *10^6^	Significant decrease with any dose(HD and LD) and duration treatment of Gentamicin	25
Sperm count (×10^6^)	Significant decrease for any dose of Gentamicin related to duration treatment	25
Sperm motility (%)	Significant decrease with high dose of Gentamicin and long-time treatment*	25
Sperm abnormality (%)	Significant decrease with high dose of Gentamicin and long-time treatment* STR has significant high percentage of sperm head defect*	25, 30
STD(Seminiferous tubule diameter)	Significant decrease with high dose of Gentamicin time-independently*	25
SE(Seminiferous epithelial height)	Significant decrease with high dose of Gentamicin time-independently *	25
CESR (×10 )	Significant decrease with gentamicin*	27, 28
Serum testosterone	Significant decrease with gentamicin*	27
LH level	Significant decrease with gentamicin*	28
MDA	Significant increase with gentamicin**	28
Sperm motility, count, and viability	Significant decrease with gentamicin**	28
SOD and catalase level	Significant decrease with gentamicin**	28
On day 3 and 4 after semen storage	Greater motility and velocity in addition of gentamicin at 15c* No significant effect on stored semen at 5c	31
Sperm motility and velocity	Decrease sperm motility and velocity after addition of gentamicin to extender* No improvement of sperm motility induced by bacteria	32

Addition of gentamicin to extender resulted in decreased motility and velocity in semen without addition of bacteria and did not improve motility parameters in semen with bacteria added

* Signiﬁcant difference compared with controls (p<0.05)

** Signiﬁcant difference compared with controls (p<0.001)

**Limitation**

There were no sufficient studies in order to assess whole aminoglycosides and fluoroquinolones drugs. In this study, 32 articles were assessed. Therefore, these results are not confidential to choose a special drug with less adverse effects on reproductive tissue and sperm parameters. Furthermore, a large number of studies had just studied gentamicin but not the other aminoglycosides. Gentamicin is a common drug of this group, so these results are not acceptable for all drugs of this group. Fluoroquinolones effect on AI was studied only in comparative study with aminoglycosides. 

## Conclusion

According to reported studies, among aminoglycosides drugs streptomycin has less negative effects on cells apoptosis and sperm parameters so this drug, is recommended instead of gentamicin and neomycin reported with more adverse effects on male reproductive tract. Furthermore, treatment dose and duration is directly related to adverse effects of gentamicin. With regard to more detrimental effects of Gentamicin, it is better to use this drug in less dosage, duration and frequency. 

Assessment of fluoroquinolones also shows these drugs have negative effects on testis tissue and sperm parameters. However, it seems in AI process using ciprofloxacinis better than gentamicin, because ciprofloxacin even in high dose is able to eliminate bacterial contamination of semen with less adverse effects on sperm function. It seems more studies are necessary in order to better compare fluoroquinolones with aminoglycosides. In other words, it should be evaluated in same parametes for fluoroquinolones and aminoglycosides. Besides, it is suggested more studies on all drugs belonging to aminoglycosides and fluoroquinolones be conducted in humans.

## Conflict of interest

The author declared that he has no conflict of interest in this study.
